# Metabolic acidosis is associated with infection severity in pediatric pyelonephritis and pneumonia

**DOI:** 10.1007/s00467-025-06708-2

**Published:** 2025-02-13

**Authors:** Hannah Brummer, Hongyue Wang, George J. Schwartz

**Affiliations:** 1 Division of Nephrology, Department of Pediatrics, University of Rochester School of Medicine and Dentistry, Rochester, NY USA; 2 Division of Nephrology, Department of Pediatrics, UBMD/Jacobs School of Medicine and Biomedical Sciences, Buffalo, NY USA; 3 Department of Biostatistics, University of Rochester School of Medicine and Dentistry, Rochester, NY USA

**Keywords:** Pyelonephritis, Pneumonia, Metabolic acidosis, *E. coli*

## Abstract

**Background:**

Pyelonephritis is the most common serious bacterial infection in pediatric patients. Several electrolyte abnormalities have been described with pyelonephritis, including metabolic acidosis. We sought to describe the frequency and clinical significance of metabolic acidosis in pediatric patients with acute *Escherichia coli* (*E. coli*) pyelonephritis, with comparison to parallel infection group of bacterial pneumonia.

**Methods:**

This was a single-center, retrospective study of pediatric patients with pyelonephritis or pneumonia hospitalization. Nadir serum bicarbonate and anion gap values were collected, and baseline and recovery values when available. Serum electrolyte and creatinine values and markers of infection severity were recorded.

**Results:**

Ninety-four pyelonephritis and 95 pneumonia subjects were included. Pyelonephritis mean nadir bicarbonate was 20 (SD 3) mEq/L, statistically significantly lower than 21 (3) mEq/L in pneumonia. Corresponding anion gap was 16 (4) mEq/L in pyelonephritis, statistically significantly lower than 17 (3) mEq/L in pneumonia. There was significant correlation between acidosis and number of fever days in pyelonephritis and between acidosis and hospital length of stay in both groups.

**Conclusions:**

This study demonstrated development of acidosis in 84% of patients with pyelonephritis and in 66% with pneumonia. The mechanism of acidosis appears to have a greater contribution from elevated anion gap in the pneumonia group compared to the pyelonephritis group. Lower nadir serum bicarbonate values are associated with longer hospital length of stay in both groups and greater number of fever days in the pyelonephritis patients.

**Graphical abstract:**

**Figure:**
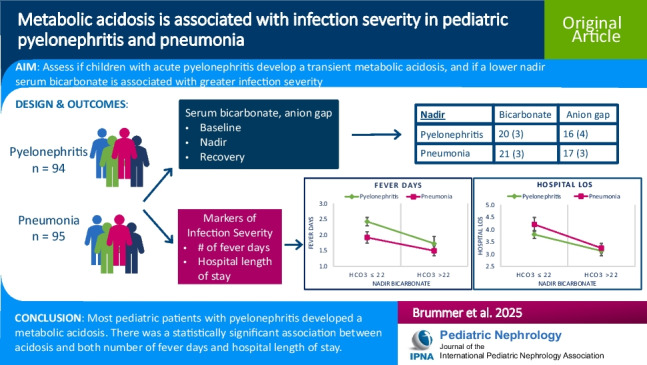
A higher resolution version of the Graphical abstract is available as Supplementary information

**Supplementary Information:**

The online version contains supplementary material available at 10.1007/s00467-025-06708-2.

## Introduction

Pyelonephritis is an infection of the kidney that often arises due to ascension of bacteria from the bladder. Acute pyelonephritis is the most common serious infection in the pediatric population, and approximately 80% of cases are caused by uropathogenic *Escherichia coli* (UPEC) [1, 2]. There is a greater risk of developing pyelonephritis in those with underlying structural urologic abnormalities, most notably vesicoureteral reflux (VUR) [1]. Repeated episodes of pyelonephritis, as well as prolonged duration of infection and inflammation, lead to permanent kidney scarring [3–5] and contribute to the development of reflux nephropathy, which accounts for up to 17% of kidney failure cases in children worldwide [6, 7].

There have been several electrolyte derangements described in pediatric cases of acute pyelonephritis, including hyponatremia, hyperkalemia, and metabolic acidosis [8–11]. The mechanism of metabolic acidosis observed in pyelonephritis has largely been attributed to an effective hypoaldosteronism [10]. Research using isolated perfused rabbit outer medullary collecting ducts demonstrated that lipopolysaccharide (LPS), which is a component of the *E. coli* outer membrane, directly inhibits bicarbonate absorption in the collecting duct, contributing to the development of metabolic acidosis [12]. It has also been shown that metabolic acidosis induced by feeding ammonium chloride-enriched chow to a mouse model prone to VUR delays clearance of UPEC, which in turn leads to increased kidney inflammation [13, 14] and greater risk of kidney scarring [1]. At this time, the prevalence and consequence of clinically significant metabolic acidosis in the setting of acute pyelonephritis in humans have not been well described. The goal of this study is to understand the frequency and potential biological significance of metabolic acidosis in pediatric patients with acute *E. coli* pyelonephritis. The underlying hypothesis is that children with acute *E. coli* pyelonephritis develop metabolic acidosis that is transient with infection treatment, and a lower serum bicarbonate is associated with increased infection severity. We also hypothesized that the acidosis in acute *E. coli* pyelonephritis is not just attributable to generalized acute infection, which was assessed by examining a separate group of children with simple pneumonia.

## Methods

This was a single-center study performed via retrospective chart review at the University of Rochester Medical Center – Golisano Children’s Hospital. Institutional Review Board approval was obtained, with exempt status due to the research holding no more than minimal risk to the subjects, and adequate provisions in place to maintain confidentiality of the data. Pediatric patients aged 2–18 years with first time hospitalization for either acute *E. coli* pyelonephritis or simple bacterial pneumonia were identified. Inclusion criteria for pyelonephritis subjects included admission from January 2012 to June 2021 for first time episode of *E. coli* pyelonephritis as determined from the electronic medical record, using pertinent ICD-9 codes 590.1, 590.11, 590.8, and 599.0, and ICD-10 codes N10 and N39.0. Urine culture was documented as growing > 100,000 CFU *E. coli*. Inclusion required a documented fever with temperature ≥ 38 C at some point during hospital admission to distinguish acute pyelonephritis from cystitis. Inclusion criteria for pneumonia subjects included admission from January 2012 to June 2021 for first time episode of pneumonia as determined from the electronic medical record, with acceptable ICD-9 codes 482.9 and 486 and ICD-10 codes J15.9 and J18.9. Patients were considered to have a diagnosis of lobar (bacterial) pneumonia if there was a documented lobar consolidation upon auscultation in the physical exam or a lobar consolidation seen on chest x-ray. Exclusion criteria for both disease groups included absence of serum bicarbonate lab values during inpatient admission, positive blood culture, positive respiratory viral panel, or positive stool PCR at the time of admission, requirement of ventilatory support, admission to the Pediatric Intensive Care Unit, clinical seizure activity during acute illness, and diabetic ketoacidosis. Exclusion criteria for pyelonephritis group included history of kidney transplant and/or urologic surgery within 14 days prior to date of admission. Patients with acute pyelonephritis could not be diagnosed with concomitant pneumonia, and vice versa.

“Day 0” was considered the date of hospital admission. Serum bicarbonate and anion gap values in both the pyelonephritis and pneumonia patient populations were collected from three distinct phases: baseline, nadir, and recovery. Baseline values, if available, were recorded when the patient was otherwise well, defined as no concurrent hospital admission and no documented infection at time of lab draw on review of the medical record. Nadir values consisted of the lowest recorded bicarbonate value and corresponding anion gap while hospitalized. Recovery values were extracted from any available outpatient blood work following hospital discharge, again with no documented concurrent infection. Metabolic acidosis was present if nadir serum bicarbonate was ≤ 22 mEq/L [15]. Blood gas data were not available for the majority of patients; thus, blood gas values were unable to be factored into classification of metabolic acidosis.

Data extracted included serum bicarbonate, anion gap, sodium, potassium, chloride, and creatinine from the nadir bicarbonate lab set, as well as from baseline and recovery time points when available. Serum bicarbonate was measured using enzymatic methodology; serum sodium, potassium, and chloride were measured by ion-selective electrode (ISE) using indirect potentiometry, and serum creatinine was measured using enzymatic methodology. Serum sodium, potassium, chloride, bicarbonate, and creatinine were measured using analyzers from Roche Diagnostics (Indianapolis, IN). Serum anion gap was calculated using the equation [serum sodium—(serum chloride + serum bicarbonate)]. High anion gap was defined as an anion gap > 16 mEq/L. Glomerular filtration rate (GFR) was estimated from serum creatinine according to the CKiD U25 equation [16]. Number of fever days, defined as days with temperature ≥ 38 C, and hospital length of stay were recorded as markers of infection severity. Saline administration was documented, and patients were considered to have saline exposure if a saline bolus was given more than 30 minutes prior to the collection of the nadir serum bicarbonate labs. Lab samples with noted hemolysis, and urine ketone values were also recorded.

Continuous variables are expressed as mean (± standard deviation) or median with interquartile range (IQR), and discrete variables are expressed as frequencies and percentages. Baseline, nadir, and recovery serum bicarbonate, anion gap, electrolytes, creatinine, and estimated GFR (eGFR) were compared between groups using two-sample t-tests. Paired t-tests were used for within group comparisons, such as baseline to nadir, and nadir to recovery, serum bicarbonate, and anion gap. Change in serum bicarbonate from baseline to nadir was compared between groups using two sample t-test. The effect of saline exposure on nadir serum bicarbonate value was assessed using a Spearman correlation coefficient. Multiple linear regression analyses were performed to evaluate the association between nadir serum bicarbonate and infection severity measures. Nadir serum bicarbonate values were subdivided into two groups (acidosis: bicarbonate ≤ 22 mEq/L, no acidosis: bicarbonate > 22 mEq/L) to assess the association between acidosis and both number of fever days and hospital length of stay, and groups were compared using Wilcoxon rank sum tests. The rate of high anion gap was compared between groups using Chi-squared test. All statistical tests were two-sided, and a *p*-value of < 0.05 was considered significant. Statistical analyses were performed using SAS 9.4 (SAS Institute, Cary, NC) and R Studio 1.4.1106 (R Studio, PBC, Boston, MA).

## Results

Ninety-four subjects with pyelonephritis and 95 subjects with pneumonia were included. The mean age of pyelonephritis subjects was 8.8 (5.6) years compared to 7.6 (4.8) years in pneumonia subjects (Table 1). Nearly all pyelonephritis patients were female, whereas there was a relatively equal sex distribution in the pneumonia patients. Within the pyelonephritis and pneumonia groups, the mean serum bicarbonate values did not significantly differ based on sex. The proportion of patients with acidosis, as defined by a nadir serum bicarbonate ≤ 22 mEq/L, also did not differ by sex within groups.

**Table 1:** Patient demographics and clinical data

	Pyelonephritis (*n* = 94)	Pneumonia (*n* = 95)	*p*-value
Age in years, mean (SD)	8.8 (5.6)	7.6 (4.8)	0.11
Sex			
Male	6 (6%)	49 (52%)	< 0.001*
Female	88 (94%)	46 (48%)	
Baseline			
*n* (% of total subjects)	25 (27%)	27 (28%)	0.78
Months from baseline to nadir labs, mean (SD)	18.4 (18.0)	16.5 (16.6)	0.68
Serum HCO_3_ in mmol/L, mean (SD)	24 (2)	25 (2)	0.02*
Serum AG in mmol/L, mean (SD)	14 (3)	14 (3)	0.57
Serum Cr in mg/dL, mean (SD)	0.46 (0.19)	0.52 (0.22)	0.32
eGFR^a^ in mL/min/1.73 m^2^, mean (SD)	105 (23)	100 (21)	0.45
Serum Na in mmol/L, mean (SD)	140 (2)	140 (2)	0.92
Serum K in mmol/L, mean (SD)	4.4 (0.5)	4.6 (0.7)	0.29
Serum Cl in mmol/L, mean (SD)	103 (2)	101 (2)	0.003*
Hospitalization (nadir labs)			
Serum HCO_3_ in mmol/L, mean (SD)	20 (3)	21 (3)	0.04*
Delta HCO_3_ in mmol/L, mean (SD)	− 4 (2)	− 3 (3)	0.02*
Serum AG in mmol/L, mean (SD)	16 (4)	17 (3)	0.008*
Delta AG in mmol/L, mean (SD)	3 (3)	3 (3)	0.77
Serum Cr in mg/dL, mean (SD)	0.57 (0.34)	0.46 (0.16)	0.006*
eGFR^a^ in mL/min/1.73 m^2^, mean (SD)	96 (41)	106 (23)	0.04*
Delta Cr in mg/dL, mean (SD)	0.17 (0.48)	0.00 (0.12)	0.09
Serum Na in mmol/L, mean (SD)	137 (3)	137 (3)	0.47
Serum K^b^ in mmol/L, mean (SD)	4.1 (0.5)	4.2 (0.5)	0.16
Serum Cl in mmol/L, mean (SD)	101 (5)	99 (4)	0.004*
Recovery			
*n* (% of total subjects)	27 (29%)	22 (23%)	0.38
Months from nadir to recovery labs, mean (SD)	22.9 (23.3)	12.4 (13.8)	0.06
Serum HCO_3_ in mmol/L, mean (SD)	24 (2)	25 (2)	0.27
Serum AG in mmol/L, mean (SD)	13 (3)	13 (2)	0.71
Serum Cr in mg/dL, mean (SD)	0.58 (0.17)	0.59 (0.17)	0.83
eGFR^a^ in mL/min/1.73 m^2^, mean (SD)	100 (24)	100 (19)	0.93

*HCO*_*3*_, bicarbonate; *AG*, anion gap; *Cr*, creatinine; *Na*, sodium; *K*, potassium; *Cl*, chloride. *: *p* < 0.05. ^a^Calculated for subjects with height recorded (for pyelonephritis and pneumonia, respectively: baseline *n* = 19, 20; nadir *n* = 81, 91; recovery *n* = 22, 18). ^b^Potassium values reported with nadir labs for *n* = 83 pyelonephritis and *n* = 83 pneumonia subjects. Remainder of potassium values hemolyzed

### Baseline

**Fig. 1:**
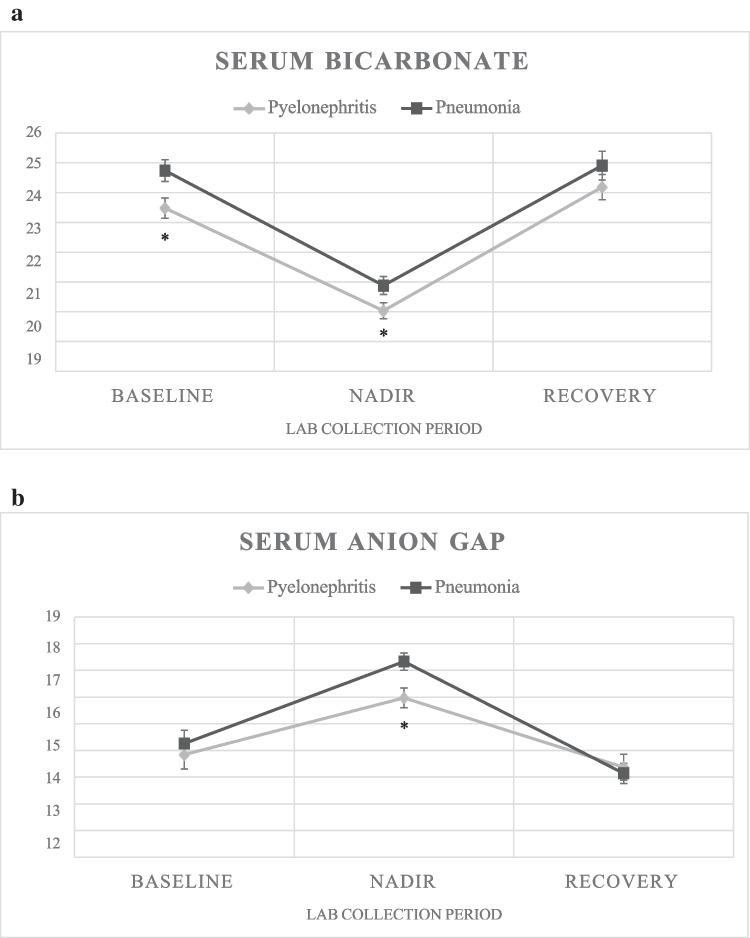
**a** Mean (standard error) serum bicarbonate values (mEq/L) at baseline, nadir, and recovery time periods in pyelonephritis and pneumonia subjects, respectively. **b** Mean (standard error) serum anion gap values (mEq/L) at baseline, nadir, and recovery time periods in pyelonephritis and pneumonia subjects, respectively. *: Statistically significant between groups, *p* < 0.05

All pyelonephritis patients had a documented urine culture growing > 100,000 CFU *E. coli*. Twenty-seven percent of pyelonephritis patients and 28% of pneumonia patients had baseline labs (see Table 1), drawn on average 18.4 months prior to hospitalization in pyelonephritis patients and 16.5 months prior in pneumonia patients. Baseline serum bicarbonate averaged (± SD) 24 (2) mEq/L with anion gap 14 (3) in pyelonephritis patients and 25 (2) mEq/L with anion gap 14 (3) in pneumonia patients (Fig. 1a, b). The difference in average baseline bicarbonate between groups was statistically significant (*p* = 0.02). There was no significant difference in baseline serum sodium or potassium. There was a significantly higher average baseline serum chloride in the pyelonephritis group (103 (2) mEq/L) compared to the pneumonia group (101 (2) mEq/L) (*p* = 0.003). Baseline serum creatinine was not significantly different between the two groups, and there was no significant difference in eGFR. Of note, a recorded baseline height for eGFR calculations was available for 19 pyelonephritis patients and 20 pneumonia patients.

### Hospitalization (nadir labs)

All pyelonephritis and pneumonia patients recovered from infection, with a median hospital length of stay of 3.5 (IQR 3,4) days in pyelonephritis patients and 3 (IQR 3,4) days in pneumonia patients. The median number of fever days was 2 (IQR 1,3) days in pyelonephritis patients and 1 (IQR 1,2) day in pneumonia patients. The lab collection containing nadir serum bicarbonate value occurred most often on hospital day 0 in both pyelonephritis and pneumonia patients.

Nearly all patients showed decreased serum bicarbonate from baseline during hospitalization, specifically 25 of 25 (100%) with pyelonephritis and 22 of 27 (81%) with pneumonia, with a significantly higher proportion in pyelonephritis compared to pneumonia (*p* = 0.03). Eighty-four percent of pyelonephritis subjects and 66% of pneumonia subjects met criteria for metabolic acidosis defined as serum bicarbonate ≤ 22 mEq/L, with the pyelonephritis group having a significantly greater proportion with metabolic acidosis (*p* = 0.005). The mean nadir serum bicarbonate value in pyelonephritis patients was 20 (3) mEq/L, which is statistically significantly lower than the mean nadir serum bicarbonate of 21 (3) mEq/L seen in pneumonia patients (*p* = 0.04) (Fig. 1a). The range of nadir bicarbonate values was 14–26 mEq/L in the pyelonephritis group and 13–27 mEq/L in the pneumonia group. The mean delta serum bicarbonate from baseline to nadir was greater in pyelonephritis patients (− 4 (2) mEq/L) compared to pneumonia patients (− 3 (3) mEq/L) (see Table 1). The mean corresponding anion gap at time of nadir bicarbonate labs was elevated in pneumonia patients (17 (3) mEq/L) compared to pyelonephritis patients (16 (3) mEq/L) (*p* = 0.008) (Fig. 1b). Forty-six percent of pyelonephritis subjects had a high anion gap, compared to 58% of pneumonia patients. The difference in these proportions was not statistically significant.

Mean serum sodium and potassium values did not significantly differ between the two groups, but there was a statistically significant higher mean serum chloride seen in pyelonephritis patients (101 (5) mEq/L) compared to pneumonia patients (99 (4) mEq/L) (*p* = 0.004). There was also a statistically significant difference in the mean serum creatinine in the pyelonephritis group (0.57 (0.34) mg/dL) compared to the pneumonia group (0.46 (0.16) mg/dL) (*p* = 0.006); however, the delta creatinine from baseline to nadir did not differ significantly between the two groups. There was a significantly greater proportion of patients in the pyelonephritis group who exhibited increase in serum creatinine from baseline to nadir lab period: 20 of 25 (80%) pyelonephritis patients, compared to 14 of 27 (52%) pneumonia patients (*p* = 0.03). A sensitivity analysis with serum creatinine outliers removed still yielded a significant difference between the two groups (*p* < 0.001).

Thirty patients in the pyelonephritis group and 11 patients in the pneumonia group had saline exposure prior to nadir bicarbonate lab draw. There was a statistically significant effect of saline exposure on the nadir bicarbonate value in the pyelonephritis group (Spearman correlation coefficient of − 0.2 mEq/L, *p* = 0.05). This effect was not statistically significant in the pneumonia group (Spearman correlation coefficient of − 0.2 mEq/L, *p* = 0.11).

The association between nadir serum bicarbonate values and the number of fever days was assessed by multiple regression analysis while controlling for confounders including hemolyzed lab sample, saline administration, and ketonuria. In the pyelonephritis group, there was a 0.8 mEq/L decrease (95% CI: − 1.4 – − 0.2) in serum bicarbonate associated with every additional day of fever. There was no significant association between nadir serum bicarbonate and number of fever days seen in pneumonia patients. There was also no significant correlation between nadir serum bicarbonate and hospital length of stay in either the pyelonephritis or pneumonia groups.

**Fig. 2:**
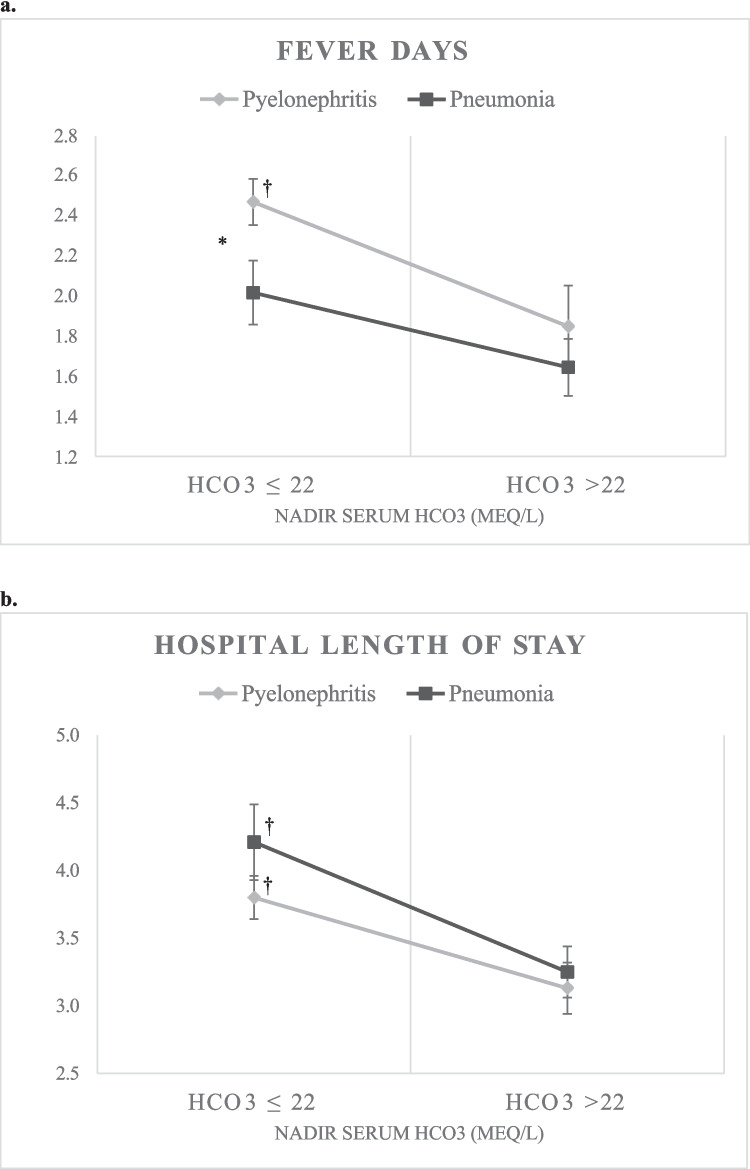
**a** Mean (standard error) fever days in patients with acidosis (HCO_3_ ≤ 22 mEq/L) vs. no acidosis (HCO_3_ > 22 mEq/L). **b** Mean (standard error) hospital length of stay in patients with acidosis (HCO_3_ ≤ 22 mEq/L) vs. no acidosis (HCO_3_ > 22 mEq/L). *:* p* < 0.05 between pyelonephritis and pneumonia disease groups. †: *p* < 0.05 within disease group. HCO_3_, serum bicarbonate; LOS, length of stay

To better characterize the role of decreased serum bicarbonate in prolonging hospitalization and fever days, subjects were divided into dichotomized groups based on nadir serum bicarbonate values, with the acidosis group representing subjects with nadir serum bicarbonate ≤ 22 mEq/L and the non-acidosis group representing subjects with nadir serum bicarbonate > 22 mEq/L. The number of fever days and hospital length of stay were evaluated based on acidosis group. In patients with pyelonephritis, those with acidosis had statistically significant greater number of fever days (median 2 (IQR 2,3) days) compared to those without acidosis (median 1 (IQR 1,2.5) days) (*p* = 0.03). This was not seen in patients with pneumonia (Fig. 2a). Pyelonephritis patients with acidosis also had a statistically significant greater number of fever days compared to pneumonia patients with acidosis (median 1 (IQR 1,2.5) days) (*p* < 0.001) (Fig. 2a). In addition, patients with acidosis had a statistically significant longer hospital length of stay compared to those without acidosis in both the pyelonephritis (median 4 (IQR 3,4) days compared to median 3 (IQR 3,3) days, respectively) (*p* = 0.04) and pneumonia (median 4 (IQR 3,5) days compared to median 3 (IQR 3,4) days, respectively) (*p* = 0.04) groups (Fig. 2b).

### Recovery

Twenty-nine percent of pyelonephritis patients and 23% of pneumonia patients had recovery labs, drawn on average 15.5 (21.9) months following hospitalization in pyelonephritis patients and 10.1 (14.1) months following hospitalization in pneumonia patients. Average recovery serum bicarbonate was 24 (2) mEq/L with anion gap 13 (3) mEq/L in pyelonephritis patients and 25 (2) mEq/L with anion gap 13 (2) mEq/L in pneumonia patients (Fig. 1a, b). The average bicarbonate and anion gap values did not differ significantly between the two groups nor did the serum creatinine (see Table 1).

## Discussion

This study demonstrated the development of metabolic acidosis in 84% of pediatric patients hospitalized for an initial episode of *E. coli* pyelonephritis. Acidosis was also demonstrated in 66% of patients with first time hospitalization for simple bacterial pneumonia, however through a potentially different mechanism with greater contribution from an elevated anion gap.

Both the pyelonephritis patients and pneumonia patients developed an acidosis by serum bicarbonate level as compared to the average serum bicarbonate values in otherwise healthy children. In children aged 12 to 18 years in the 2017–2020 pre-pandemic NHANES cohort [17], the average serum bicarbonate was 25 (2) mEq/L, with the 5th percentile 21 mEq/L and 95th percentile 28 mEq/L. This value of 25 mEq/L corresponds with the average baseline bicarbonate value seen in pneumonia patients (25 mEq/L) and the average recovery bicarbonate values seen in both pyelonephritis and pneumonia patients (24 and 25 mEq/L, respectively). The average baseline bicarbonate value in pyelonephritis patients was slightly lower, which could be accounted for by underlying predisposition to acidosis from a history of hydronephrosis, vesicoureteral reflux, or underlying kidney damage. Within the pyelonephritis group, there was some degree of hydronephrosis documented on kidney ultrasound in 10 patients and some degree of VUR documented on voiding cystourethrogram in 17 patients.

The NHANES dataset also revealed an average serum anion gap value of 14 (2) mEq/L in healthy teenagers aged 12 to 18 years, with 5th percentile 10 mEq/L and 95th percentile 18 mEq/L. The cutoff value to be considered an elevated anion gap at our institution’s laboratory is > 16 mEq/L, which is approximately 1 SD above the calculated NHANES average. The average anion gap value from pneumonia patients from the nadir bicarbonate lab set was elevated at 17 mEq/L, compared to the average anion gap value of 16 mEq/L in pyelonephritis patients. This suggests that the underlying mechanism of acidosis seen in pneumonia patients may be different compared to pyelonephritis, as the pneumonia patients seem to have a greater anion gap contribution. This anion gap contribution does not appear to be due to antibiotic dosing, as the anion composition of the commonly used antibiotics is estimated to contribute at most about 0.27 mmol/L to the serum anion gap level [18].

Serum chloride values were significantly higher at both baseline and nadir in the pyelonephritis group compared to the pneumonia group. An elevated serum chloride along with lower serum bicarbonate is consistent with hyperchloremic metabolic acidosis in pyelonephritis patients. This again supports a different, normal anion gap acidosis mechanism in pyelonephritis patients as compared to the overall high anion gap acidosis seen in pneumonia patients. Serum sodium and potassium values did not differ significantly between groups in both the baseline and nadir lab periods, and the mean values were within normal range. This rules against an effective aldosterone resistance or pseudohypoaldosteronism as the underlying etiology for metabolic acidosis seen in pyelonephritis patients, as one would expect to see a concomitant hyperkalemia and/or hyponatremia. Serum creatinine values were significantly higher, and corresponding eGFR significantly lower, during hospitalization in pyelonephritis patients as compared to pneumonia patients due to the presumed kidney parenchymal inflammation. There was no significant difference in baseline or recovery serum creatinine or eGFR between the two groups, indicating recovery from the presumed first episode of pyelonephritis. This study was limited to first-time pyelonephritis hospitalizations, but with repetitive episodes of pyelonephritis and associated acute kidney injury, it is anticipated there would be a lesser degree of subsequent recovery and thus development of permanent damage.

The serum bicarbonate threshold used to define metabolic acidosis was based on findings from pediatric patients with chronic kidney disease, which showed an association between greater progression of glomerular chronic kidney disease and serum bicarbonate values ≤ 22 mEq/L [15]. This threshold is > 1 SD lower than the NHANES average serum bicarbonate of 25 mEq/L. Current clinical practice often is not to treat metabolic acidosis until serum bicarbonate is below 18 mEq/L, but nadir serum bicarbonate ≤ 22 mEq/L was associated with a greater number of fever days in pyelonephritis patients, and a greater hospital length of stay in both pyelonephritis and pneumonia patients. Data from this study suggest it may be beneficial to treat with alkali supplementation or to use more balanced IV fluid solutions such as lactated ringers or plasmalyte as opposed to saline derivatives, for milder bicarbonate derangements. Future prospective studies may explore if alkali therapy leads to decreased infection severity.

Strengths of this study included large sample size, examination of uncomplicated illness using localized bacterial pyelonephritis or pneumonia, and the assessment of other markers including kidney function and electrolyte derangements. Limitations include the study being single center and the retrospective nature of the study; however, other sources of acidosis were controlled for to the greatest extent possible through exclusion criteria and adjustment for confounders in data analysis. It was difficult to extrapolate the degree of certain sources of acidosis through chart review, namely, presence and degree of diarrhea and respiratory distress including tachypnea. The pyelonephritis group was predominately female, compared to more even split by sex in the pneumonia group; however, data analysis did not reveal any significant discrepancies in nadir bicarbonate values by sex within either group. GFR estimates were limited by a lack of recorded height for several patients. Additionally, there were several statistically significant differences in certain blood analytes; however, these differences may not be considered clinically significant in practice. Another limitation was the use of serum bicarbonate as a marker of metabolic acidosis and the lack of blood gas analyses. Blood gas data were only available for three pneumonia patients and zero pyelonephritis patients. Prospective studies involving blood gas data would be instrumental in better elucidating the acid–base status. It is also difficult to determine if those with pyelonephritis and acidosis had more severe infection than those without acidosis; that is, was the acidosis simply a marker of more severe generalized infection and inflammation?

## Conclusion

This study demonstrated the development of acidosis in 84% of pediatric patients with pyelonephritis and 66% of those with pneumonia. Acidosis, as evidenced by average nadir serum bicarbonate, was more severe in pyelonephritis patients compared to pneumonia patients, and the pneumonia group had a larger anion gap contribution to the acidosis. There was a significant association between acidosis and increased number of fever days in pyelonephritis patients and increased length of stay in both pyelonephritis and pneumonia patients. Prospective studies are needed to better delineate the acid–base status of these patients and the relationship between acidosis and degree of general infection severity.

## Supplementary Information

Below is the link to the electronic supplementary material.

Graphical abstract (PPTX 81 KB)

## Data Availability

The datasets generated during and/or analyzed during the current study are available from the corresponding author on reasonable request. The NHANES 2017–2020 Pre-Pandemic Laboratory Data analyzed and referenced in the “Discussion” portion of the study is publicly available and accessible at: https://wwwn.cdc.gov/Nchs/Nhanes/Search/DataPage.aspx?Component=Laboratory&Cycle=2017-2020.
