# Supplementary material for: Metabolic acidosis is associated with infection severity in pediatric pyelonephritis and pneumonia
Source: Pediatr Nephrol. 2025 Feb 13;40(7):2277–85. doi: 10.1007/s00467-025-06708-2 (PMC12116824; doi:10.1007/s00467-025-06708-2)
Supplement: Supplementary file 1 — Graphical abstract (PPTX 81 KB) [file 467_2025_6708_MOESM1_ESM.pptx]

## Slide 1
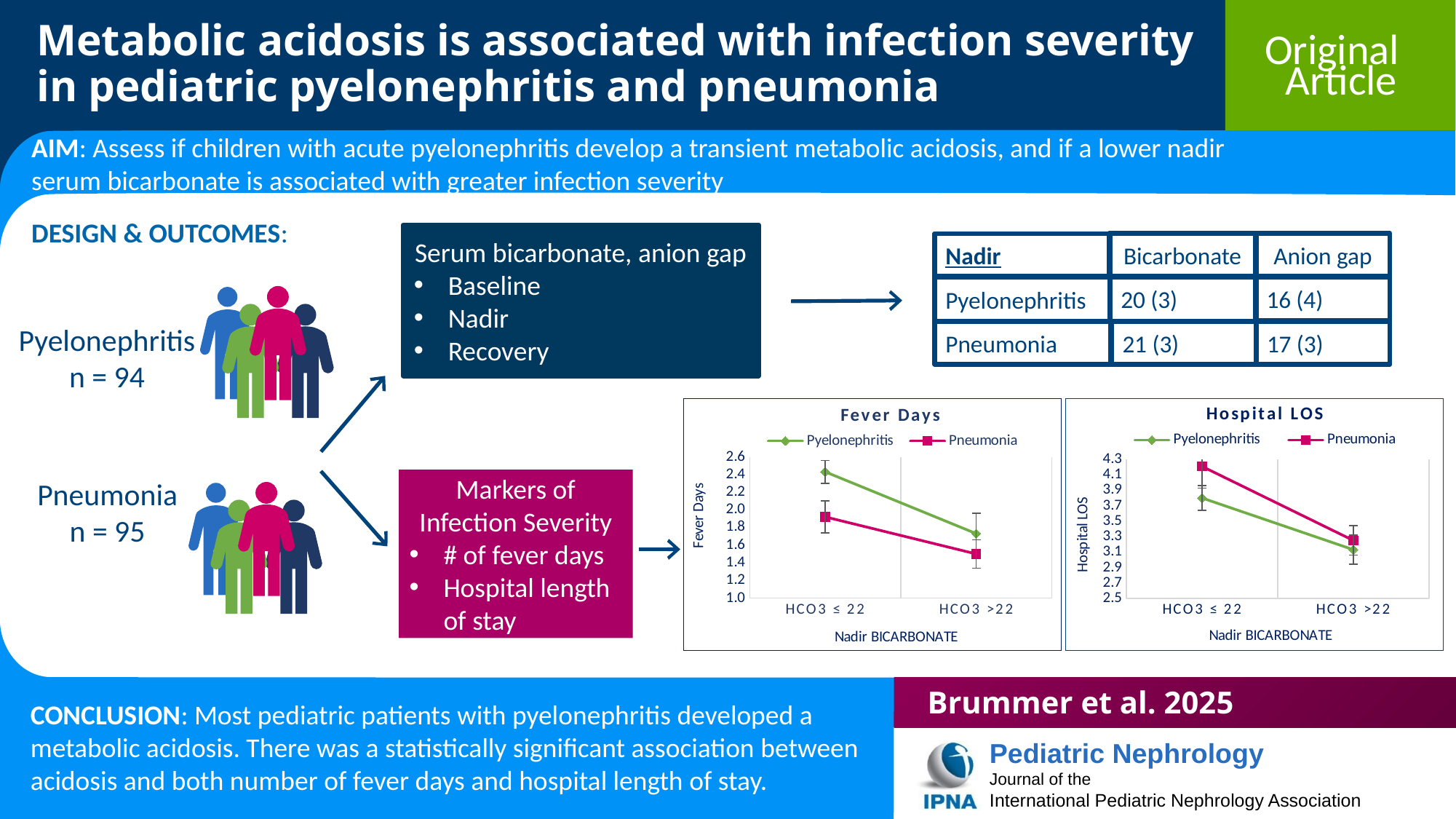

Metabolic acidosis is associated with infection severity in pediatric pyelonephritis and pneumonia
AIM: Assess if children with acute pyelonephritis develop a transient metabolic acidosis, and if a lower nadir serum bicarbonate is associated with greater infection severity
DESIGN & OUTCOMES:
Serum bicarbonate, anion gap
Baseline
Nadir
Recovery
Anion gap
Bicarbonate
Nadir
16 (4)
20 (3)
Pyelonephritis
17 (3)
Pneumonia
21 (3)
Pyelonephritis
n = 94
### Chart: Fever Days
| Category | Pyelonephritis | Pneumonia |
|---|---|---|
| HCO3 ≤ 22 | 2.43 | 1.92 |
| HCO3 >22 | 1.73 | 1.5 |
### Chart: Hospital LOS
| Category | Pyelonephritis | Pneumonia |
|---|---|---|
| HCO3 ≤ 22 | 3.8 | 4.21 |
| HCO3 >22 | 3.13 | 3.25 |Markers of Infection Severity
# of fever days
Hospital length of stay
Pneumonia
n = 95
Brummer et al. 2025
CONCLUSION: Most pediatric patients with pyelonephritis developed a metabolic acidosis. There was a statistically significant association between acidosis and both number of fever days and hospital length of stay.
